# Supplementary material for: Sample size requirements for genetic studies on yellowfin tuna
Source: PLoS One. 2021 Nov 4;16(11):e0259113. doi: 10.1371/journal.pone.0259113 (PMC8568148; doi:10.1371/journal.pone.0259113)
Supplement: Attachment — Submitted filename: Comments to the Author.doc [file pone.0259113.s003.doc]

**Comments to** **the Author:**

In this work, authors assessed the numbers of individual yellowfin tuna (*Thunnus albacares*) and genetic markers required for ocean-basin scale inferences, and they assessed this for three distinct data analysis tasks: testing for differences between genetic profiles; stock delineation, and assignment of individuals to stocks. The results obtained in this work can help designers of molecular ecological surveys for yellowfin tuna to assess whether the information content is adequate for the required inferential task. This work is interesting and very meaningful for researchers. The figures are well presented in the manuscript. Overall, the article deserves publication. However, I also found some shortcomings that require revisions as specified below.

1. There are too many keywords, I think 5-6 keywords are OK. For example, “Single Nucleotide Polymoprhism” is the same with “SNP”.
2. Line 8-9: The first sentence is not very important in the abstract, and it is suggested to delete.
3. In this article, the authors used italics in some sentences, I don’t know the meaning. Such as, “and” in Line 19, “uncertain” in Line 31, “re-sampled” in Line 64, “increasing” in Line 280, and so on.
4. Line 89-90: In order to make it more understandable to readers, it is suggested to mark these four locations in the world map and add this figure to the manuscript.
5. In the Methods section, there is no need to describe the method and the reason so detailedly. For example, Line 99-103, Line 121-125, these parts can be removed to the discussion section. Besides, in the section 2.3 “Statistical Analyses”, is it necessary to depicted it in so detail? The authors can check all the manuscript, and make the Methods and Results sections more succinctly and understandable.
6. In the manuscript, some sentences are not professional and succinctly. For example, “Some researchers may be familiar with this idea as” in Line 149. Please check all the manuscript, and make it more succinctly and professional.
7. In Line 405, delete one “.” after performed.
8. Please check the format of references. For example, “2013, 05” in Line 416, “2012, 09” in Line 446, “;” in Line 454.
