# Supplementary material for: Sample size requirements for genetic studies on yellowfin tuna
Source: PLoS One. 2021 Nov 4;16(11):e0259113. doi: 10.1371/journal.pone.0259113 (PMC8568148; doi:10.1371/journal.pone.0259113)
Supplement: Attachment — Submitted filename: Response2.docx [file pone.0259113.s004.docx]

Response to the Review of Manuscript PONE-D-21-20524: “Sample Size Requirements for Genetic Studies on Yellowfin Tuna”

Scott D. Foster, Pierre Feutry, Peter Grewe and Campbell Davies

XXth August 2021

# **General Remarks**

We would like to thank the reviewers and editor for their critique of our manuscript. We are happy to be given the opportunity to provide a revised manuscript that addresses the concerns raised. The manuscript is now better for the revision.

Each comment from each of the reviewers will be responded to in turn. The reviewers’ comments are typeset in *italics* and our responses are in plain text.

# **Reviewer 1**

*General comment: In my personal opinion, this paper is not useful and helpful for the community at all for different reasons:*

1. *It is well established that working with big number of markers (>1K) in population genomics the dataset is not sensitive at all to the number of individuals sampled (Aguirre-Liguori et al 2020). It is clear that the authors are used to work with microsatellite dataset.*

   Whilst our study supports the claim that datasets with more than 1K markers have increased power, we note that we provide data where this simply is not true (e.g. Figure 2 in the original submission, which is Figure 3 in the revision). In that figure, the small genetic differentiation means that the extra information from more markers, or more individuals, is necessary. So, this work sheds new light on how “well established”, or perhaps how widely applicable, this 1K rule is. In short, it is not applicable everywhere and for all purposes.

   In any case, our study does not just look at the number of markers required. It looks at how the number of fish *and* how the number of markers interact to determine the sample size requirements. Further, it does this for a set of statistical analyses that are relevant to fisheries managers. In some senses, we run in parallel to the recent work of Aguirre-Liguori et al. (2020) who concentrate of point estimates of genetic dissimilarity and landscape measures.

   We are grateful for the pointer to the Aguirre-Liguori et al. (2020) reference. It had previously escaped our attention as sometimes very recent work does – we were already well-progressed when it was published. We have added it to our references and have referred to similarities/differences in methods and results where appropriate in the methods section and in the discussion.
2. *The field is rapidly moving from restriction site-associated DNA sequencing to low-coverage Whole Genome Resequencing and this is increasing even more our capability to scan the genomes and to obtain more loci and markers. These methods allow to infer complex models of population history even if small sample sizes are available (i.e. 10-15 individuals).*

   This is almost certainly true. However, that does not mean that all future studies will be using new technologies. There will be a place for the information contained in this manuscript for many years to come. We have added a paragraph to this effect in the discussion.

   We also note that, even if this were true, it does not make the work in our manuscript wrong or misleading. It just means that the readership and perhaps even the utility of the findings are limited.
3. *For pelagic fish species, it is very complicated to obtain large number of samples. So it Is absolutely useless to point out those numbers for this species. In fact in their paper on the population genomics of yellowfin tuna in the Pacific (Grewe et al., 2015) they have around 15-20 samples per sampling location.*

   We agree that large sample sizes for pelagic fish can be difficult to obtain. But it is not impossible. We do not share the the reviewer’s opinion that it is useless to point out these sample size requirements. The results presented in this paper are honest and factual. They will help managers understand the requirements of genetic studies, and it will help them understand how information from existing studies should be weighted and evaluated. The results will aid scientists plan upcoming surveys and laboratory work. All this has already been highlighted in the Discussion.

   In our previous work, (Grewe et al.; 2013) we have used smaller sample sizes. That work was performed before we had access to this information. We performed that work using the best available information at the time, which was scant for yellowfin tuna. That study was one of the reasons that this one was undertaken.

   We already have a paragraph in the Discussion, the concluding paragraph no less, that explains the importance of sample size but in the context of practical considerations. In short, small sample sizes can provide some information where none is previously available. That does not mean however, that small sample sizes should be planned for.
4. *It is also complete pointless to have different sample sizes for different task when we are now dealing with more than thousands of markers and we can map them to the genome of many non reference species.*

   We disagree. There is utility in having different sample sizes for different statistical tasks. If we took a one-size fits-all approach then the recommendation would have to be based on the most conservative situation (assignment), which is beyond the resources of many projects and is also (arguably) a less common inferential goal.

   We appreciate that the development of mappings between dense representations of different species’ genomes is an exciting prospect. However, for many species, including many tunas, there still isn’t a widely-available mapped genome let alone a mapping to other species. So, while it is exciting, it is also not practical at the current point in time.

*For all these reasons, I would suggest to reject this paper!*

It is a shame that the reviewer feels this way. We disagree. To us, none of these comments indicate that this work is done poorly or even inadequately. The comments are the opinion of the reviewer and consider the context of the manuscript and its utility. We do not feel that the logical conclusion from these comments is ‘rejection’.

# **Reviewer 2**

*In this work, authors assessed the numbers of individual yellowfin tuna (Thunnus albacares) and genetic markers required for ocean-basin scale inferences, and they assessed this for three distinct data analysis tasks: testing for differences between genetic profiles; stock delineation, and assignment of individuals to stocks. The results obtained in this work can help designers of molecular ecological surveys for yellowfin tuna to assess whether the information content is adequate for the required inferential task. This work is interesting and very meaningful for researchers. The figures are well presented in the manuscript. Overall, the article deserves publication. However, I also found some shortcomings that require revisions as specified below.*

Thanks for the positive, yet qualified, comments. Upon reflection, and with these comments, we agree with them.

1. *There are too many keywords, I think 5-6 keywords are OK. For example, “Single Nucleotide Polymoprhism” is the same with “SNP”.*

   The number of keywords has been reduced substantially. We have also added them to the body of the manuscript, and will update the list within the submission system too.
2. *Line 8-9: The first sentence is not very important in the abstract, and it is suggested to delete.*

   We can see how it could be considered to be superfluous, and have therefore removed it. We have also added a few contextual words to the second sentence.
3. *In this article, the authors used italics in some sentences, I don’t know the meaning. Such as, “and” in Line 19, “uncertain” in Line 31, “re-sampled” in Line 64, “increasing” in Line 280, and so on.*

   These italics, and others, have been removed.
4. *Line 89-90: In order to make it more understandable to readers, it is suggested to mark these four locations in the world map and add this figure to the manuscript.*

   A new figure has been added (with label of “Figure 2” in revised manuscript).
5. *In the Methods section, there is no need to describe the method and the reason so detailedly. For example, Line 99-103, Line 121-125, these parts can be removed to the discussion section. Besides, in the section 2.3 “Statistical Analyses”, is it necessary to depicted it in so detail? The authors can check all the manuscript, and make the Methods and Results sections more succinctly and understandable.*

   We have edited this section with the aim to make it as easily understood as possible. Whilst editing however, we have also tried to balance this interpretability with providing sufficient detail for readers to re-create the research. We have not moved any material to the discussion: if not appropriate for the methods section, then it is not appropriate anywhere.
6. *In the manuscript, some sentences are not professional and succinctly. For example, “Some researchers may be familiar with this idea as” in Line 149. Please check all the manuscript, and make it more succinctly and professional.*

   We have re-read and edited the manuscript.
7. *In Line 405, delete one “.” after performed.*

   Removed
8. *Please check the format of references. For example, “2013, 05” in Line 416, “2012, 09” in Line 446, “;” in Line 454.*

   Checked and corrected.
